# Spinous Process Combined With a Titanium Mesh Cage as a Bone Graft in the Stability Reconstruction of Lumbar or Lumbosacral Spinal Tuberculosis

**DOI:** 10.3389/fsurg.2022.818926

**Published:** 2022-04-04

**Authors:** Hongqi Zhang, Lige Xiao, Mingxing Tang, Guanteng Yang

**Affiliations:** ^1^Department of Spine Surgery and Orthopaedics, Xiangya Hospital, Central South University, Changsha, China; ^2^National Clinical Research Center for Geriatric Disorders, Xiangya Hospital, Central South University, Changsha, China

**Keywords:** titanium mesh cage, spinous process bone graft, spinal tuberculosis, posterior-only approach, intervertebral bone grafting

## Abstract

**Background:**

Autogenous bone grafts, such as iliac bone or rib struts, have been used in the anterior reconstruction of spinal tuberculosis (STB) and have their own benefits and limitations. Here, we introduced a new method, the spinous process (SP), combined with a titanium mesh cage (TMC) as a bone graft in the stability reconstruction of lumbar or lumbosacral STBs. By retrospectively comparing patients who received SP+TMC to traditional TMC bone grafts or allogeneic bone grafts in terms of safety, efficacy and cost-effectiveness, we aimed to evaluate whether SP+TMC could be a possible alternative method.

**Methods:**

From 2010 to 2018, 69 patients who underwent one-stage posterior debridement with grafts and internal fixation within a single lumbar or lumbosacral segment were included in this study. Twelve patients who received SP combined with a TMC (SP+TMC, group A), 30 patients who received a TMC only (group B), and 27 patients who received allografts (group C) were included. Measurements including operative time, blood loss, length of hospital stay, visual analog scale (VAS) score, Oswestry Disability Index (ODI), erythrocyte sedimentation rate (ESR), C-reactive protein (CRP), American Spinal Injury Association Impairment (ASIA) grade, final follow-up (FFU) duration and postoperative complications were recorded. Radiological measurements, including the number of segments fixated, the number of pedicle screws used, the Cobb angle, pelvic parameters, and the bony fusion time, were reviewed. All outcomes were analyzed using SPSS 25.

**Results:**

We found that the SP+TMC group had fewer fixation segments, fewer pedicle screws implanted, a shorter operative time, reduced blood loss, and a considerably lower hospital cost than allografts. In addition, the TMC group had a comparable clinical outcome with the TMC group regarding lower economic cost.

**Conclusion:**

Our study demonstrates that compared to a TMC or allograft, the use of SP combined with a TMC as a bone graft is an effective and reliable approach for the surgical management of one-level lumbar or lumbosacral spinal tuberculosis, leading to effective restoration of spinal stability. Furthermore, this approach is a cost-effective structural bone grafting method, especially for patients in developing countries.

## Introduction

Tuberculosis is a major health problem worldwide, with an estimated 10.0 million new cases each year (1). Bone tuberculosis is the most common type of extrapulmonary tuberculosis, and spine tuberculosis (STB) accounts for 50% of all bone tuberculosis cases, with no age or sex exempt from spinal TB (2). Among all spinal regions, the lumbar and lumbosacral segments support the majority of body weight and exhibit the greatest mobility, resulting in chronic damage and thus increasing susceptibility. Hence, the lumbar region is the most frequently affected site in 38.2–59.57% cases of STBs, while 8.0–8.48% cases of STBs involve lumbosacral segments (3, 4). As the onset of disease can be insidious and difficult to diagnose early, patients' initial symptoms can include minor back pain and the development of kyphotic deformities with/without neurological complications. With limited health care resources, a large number of patients from developing countries are seen for the first time at an advanced stage of disease (1). The kyphotic deformity ranges from mild knuckle-shaped deformation to angular or rounded kyphotic deformity. Paraplegia, the most dreaded complication, occurs in 10% to 30% of those patients (4).

Surgical treatment plays a key role in the management of patients with STB who present with spinal deformity, severe or progressive neurologic dysfunction, spinal instability, extensive paravertebral, and epidural abscess (5). With the introduction of the spinal pedicle screw system, a one-stage posterior approach has been increasingly adopted by surgeons to treat lumbar and lumbosacral STBs (6–8). However, the spinal pedicle screw system only provides temporary stability, with long-term stability primarily relying on bony fusion of the vertebral defect. At present, the most commonly used bone grafts for STB surgery are allogeneic bone grafts (allografts), autogenous iliac bone grafts, and titanium mesh cages (TMCs) filled with allogeneic or autogenous bone (9, 10), each of which has benefits and limitations. The autogenous iliac bone graft is considered the gold standard due to its high bone fusion rate, but it may result in additional surgical trauma and complications at the donor site (10, 11). Other autogenous bone methods, such as rib strut or spinous process (SP) and transverse process (TP) bone, have also been applied in one-level thoracic or lumbar tuberculosis (12–14). For surgery using the posterior approach, the SP is spontaneously exposed during this process, reducing operative time, bleeding, and trauma. Additionally, SP as an autogenous bone graft benefits osteogenesis, bone healing, bone conduction, and osteoinduction since it effectively fills the defect space. However, regarding structural strength and stability, the SP is not as effective as a TMC. Considering this, we decided to use SP combined with a TMC (filled with autogenous cancellous bone granules) for anterior reconstruction.

To date, no study has reported the use of SP combined with a TMC as a bone graft in the surgical treatment of lumbosacral STB. By retrospectively comparing patients who received SP+TMC to those who received traditional TMC bone grafts or allogeneic bone grafts in terms of safety and efficacy, we aimed to evaluate whether SP+TMC could be a possible alternative method for surgeons. Moreover, since patients in less developed areas are more likely to be affected by STB, individual hospital costs were also reviewed.

## Materials and Methods

### Patients

Patients with lumbar and lumbosacral STB who were hospitalized and underwent one-stage posterior focus debridement, interbody graft, posterior instrumentation, and fusion surgery in our department from January 2010 to February 2018 were included in this study. The patients were required to meet all of the following inclusion criteria: (1) the level involved was limited from L1 to S1; (2) only one segment was involved, or multiple segments were involved, but only one level needed surgical intervention; (3) no evidence of extensive TB abscess was observed; (4) the focal tissue was expected to be completely debrided via the posterior approach only; and (5) syndromes including spinal instability, vertebral collapse, kyphosis deformity, bone destruction, spinal cord compression, or progressive neurological impairment were observed. Patients presenting with any of the following conditions were excluded: (1) multilevel lesions needing surgical intervention; (2) deep multiple cold abscesses or an abscess that was primarily localized in the anterior column, which might be beyond the ability of debridement via the posterior approach; (3) other types of spinal disease or a history of spine surgery; and (4) active TB or other contraindications. This study was approved by our hospital and was conducted following the Declaration of Helsinki. All participants signed the informed consent. The benefits and limitations of each method were fully explained to the patients and their relatives before surgery to allow the patients to decide their preferred method of treatment.

### Preoperative Management

All patients enrolled in this study received routine anti-tuberculosis chemotherapy (HREZ4) for 2–4 weeks. Supportive nutritional therapy was administered to rectify hypoproteinaemia and anemia. Related indexes, such as ESR and CRP, were closely monitored. In all patients, ESR was strictly controlled below 40 mm/h, except for one patient who experienced progressive paralysis during presurgical chemotherapy.

### Surgical Procedure

The patients were in the prone position after administration of general endotracheal anesthesia. A midline incision was made to expose posterior spinal elements of vertebrae that were 1–2 levels superior and inferior to the infected segment. After locating the infected vertebrae using C-arm fluoroscopy, the entire SP was cut off using spinal scissors and then preserved in clean wet gauze for future use. Posterior pedicle screws were allowed to be used in the affected vertebrae when necessary. A temporary rod on the mild side of the focus was installed to stabilize the spine. Unilateral facetectomy and a laminectomy were performed on the focal side. The nerve root and the dura mater were pulled to expose the infected intervertebral space under the protection of the nerve root retractor. Debridement was performed via a posterolateral approach to vertically remove the collapsed vertebrae and necrotic intervertebral disk. Focal lesions were removed under direct visualization, while contralateral lesions were scraped until the surface of the sclerotic bone turned into bleeding subhealthy bone tissue using a long curette at multiple angles. A suitable flush tube was plunged in to wash the cavity with hydrogen peroxide and saline. For patients treated with TMC+SP, we suitably trimmed the TMC and SP depending on the remaining space before implantation; typically, we first implanted the shared SP followed by the TMC. For patients treated with TMCs, one or more TMCs were appropriately trimmed and then implanted according to the space of the bone graft area. Similarly, for patients treated with allografts, the surgeon will shape the allogeneic iliac bones according to the size of the bone graft area and then implant them. The titanium rod was tightened with proper pressure, and the TMC and SP were confirmed to be in good position. Streptomycin and isoniazid were locally administered. The vertebral lamina and the small joints were reconstructed afterwards. A drainage tube was placed heading to the specially formed TMC. The incision was closed by layer.

### Postoperative Care

The drainage tube was removed when the volume of drainage was <20 ml per day. Anti-TB therapy was continued for 12-18 months. ESR, CRP, and liver function were followed up each month, while X-rays and CT were performed to evaluate spinal status. Postoperative rehabilitation guidance was performed 1 week after surgery. Patient follow-up (FU) was recommended at 3 months, 6 months, 1 year, and then annually after surgery.

### Outcome Assessment

#### Demographic Data

The following demographic data were collected from each patient: age, sex, residence, occupation, annual individual income in USD, and infected spinal level.

#### Clinical Assessments

For all patients, the following indexes were recorded at each timepoint (preoperative, before discharge, and at FFU): patient residence and income, average operation time, blood loss, hospital stay and cost, VAS score, ODI, ASIA grade, ESR and CRP.

#### Radiological Assessments

(1) Fixation segment: fusion of one disc is considered to be one fixation segment; (2) number of pedicle screws; (3) Cobb angle in the sagittal plane: the angle between the upper endplate and the inferior endplate of the lesion vertebral body in the sagittal lane is defined as the Cobb angle in our study; (4) pelvic parameters: pelvic tilt (PT), pelvic incidence (PI), sacral slope (SS), lumbar lordosis (LL), and PI-LL; (5) bone grafting fusion: bone graft fusion was assessed using the radiologic criteria reported by Bridwell et al. (15).

### Statistical Analysis

The results were recorded and analyzed using SPSS software version 25.0 (SPSS Inc., Chicago, IL). Quantitative data are expressed as the mean ± standard deviation. ANOVA was used for intergroup comparisons of quantitative data, and paired *t* tests were used for intragroup comparisons. The chi-square test was performed for intergroup comparisons of nonnormally distributed qualitative data. For normally distributed qualitative data, the Wilcoxon rank sum test and Mann–Whitney rank sum test were used (intragroup and intergroup, respectively). *P* < 0.05 was considered a significant difference.

## Results

**Figure 1:**
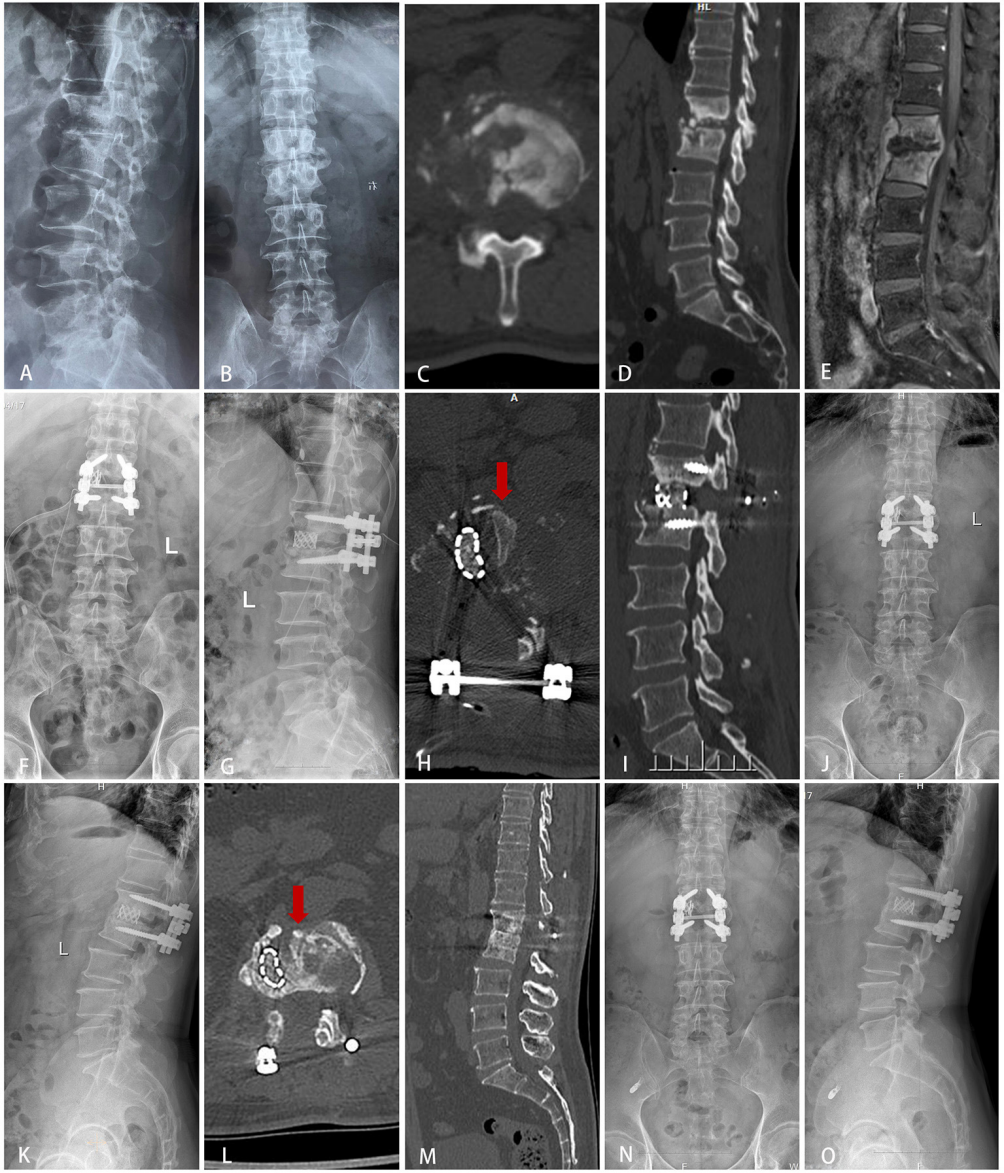
Typical cases of group A (SP+TMC bone graft). A 63-year-old male was diagnosed with tuberculous spondylitis after an eight-month history of severe back pain. The infection had been resistant to chemotherapy for 4 months. **(A–E)** Preoperative X-ray, MRI and CT showed that the lesion around the vertebral body of L1/2 developed an abscess with marked bony destruction. The abscess involved in the spinal canal with cord compromise resulted in neurologic deficits. **(F–J)** Postoperative X-ray and CT showed complete resolution of the epidural abscess and decompression of the neural component. Interbody grafts using titanium mesh cages and spinous processes were placed satisfactorily. **(K–O)** Final follow-up (2 years) radiographs showed good bone fusion.

**Figure 2:**
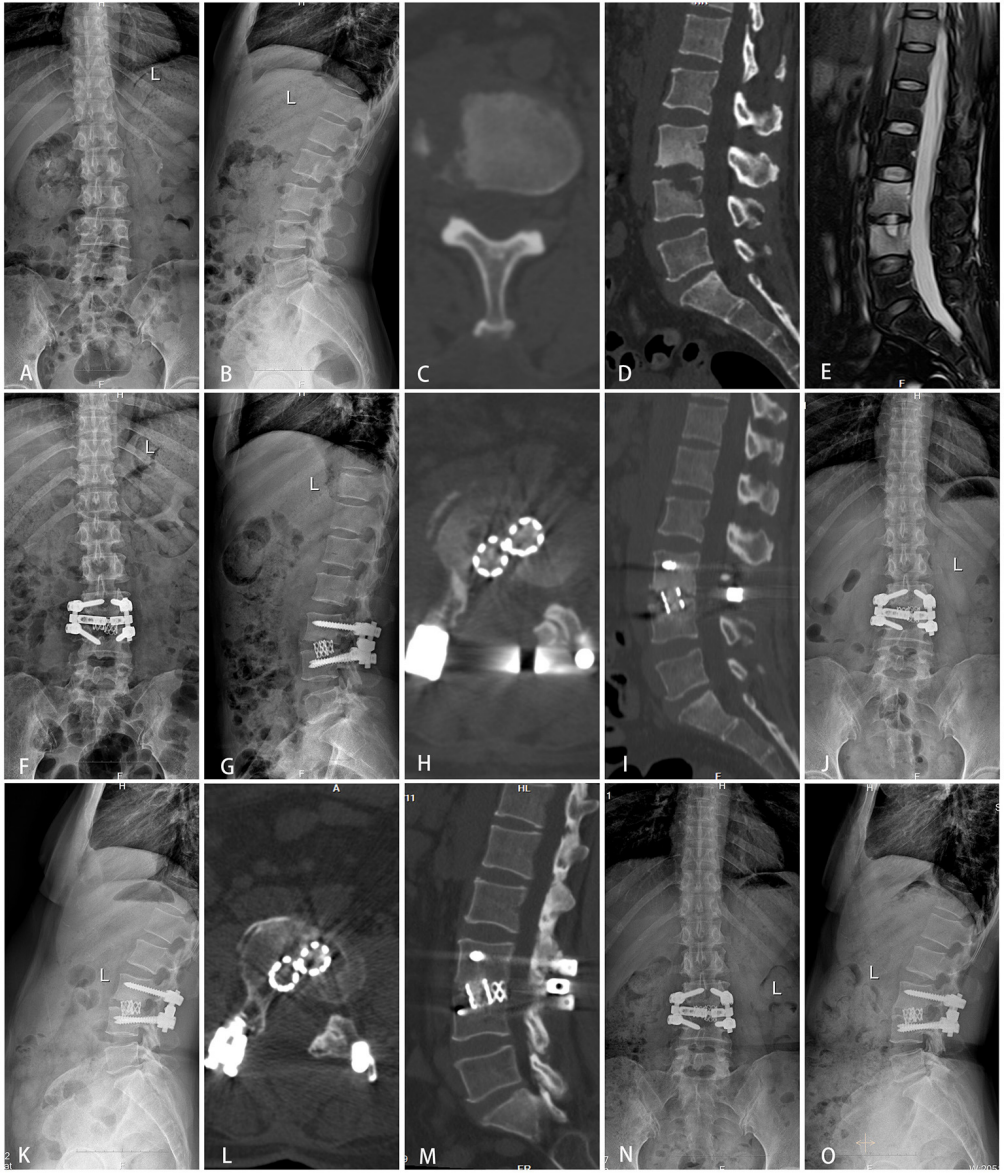
Typical cases of group B (TMC bone graft). A 49-year-old female was diagnosed with tuberculous spondylitis after a six-month history of low back pain. The infection had been resistant to chemotherapy for 1 month. **(A–E)** Preoperative X-ray, MRI and CT showed that the lesion around the vertebral body of L3/4 developed an abscess with marked bony destruction. **(F–I)** Postoperative X-ray and CT showed complete resolution of the epidural abscess and decompression of the neural component. Interbody grafts using two titanium mesh cages were placed satisfactorily. **(J–M)** One year follow-up showed good bone fusion. **(N,O)** Final follow-up (2 years) radiographs showed good bone fusion and no obvious displacement or subsidence of the titanium mesh cage.

**Figure 3:**
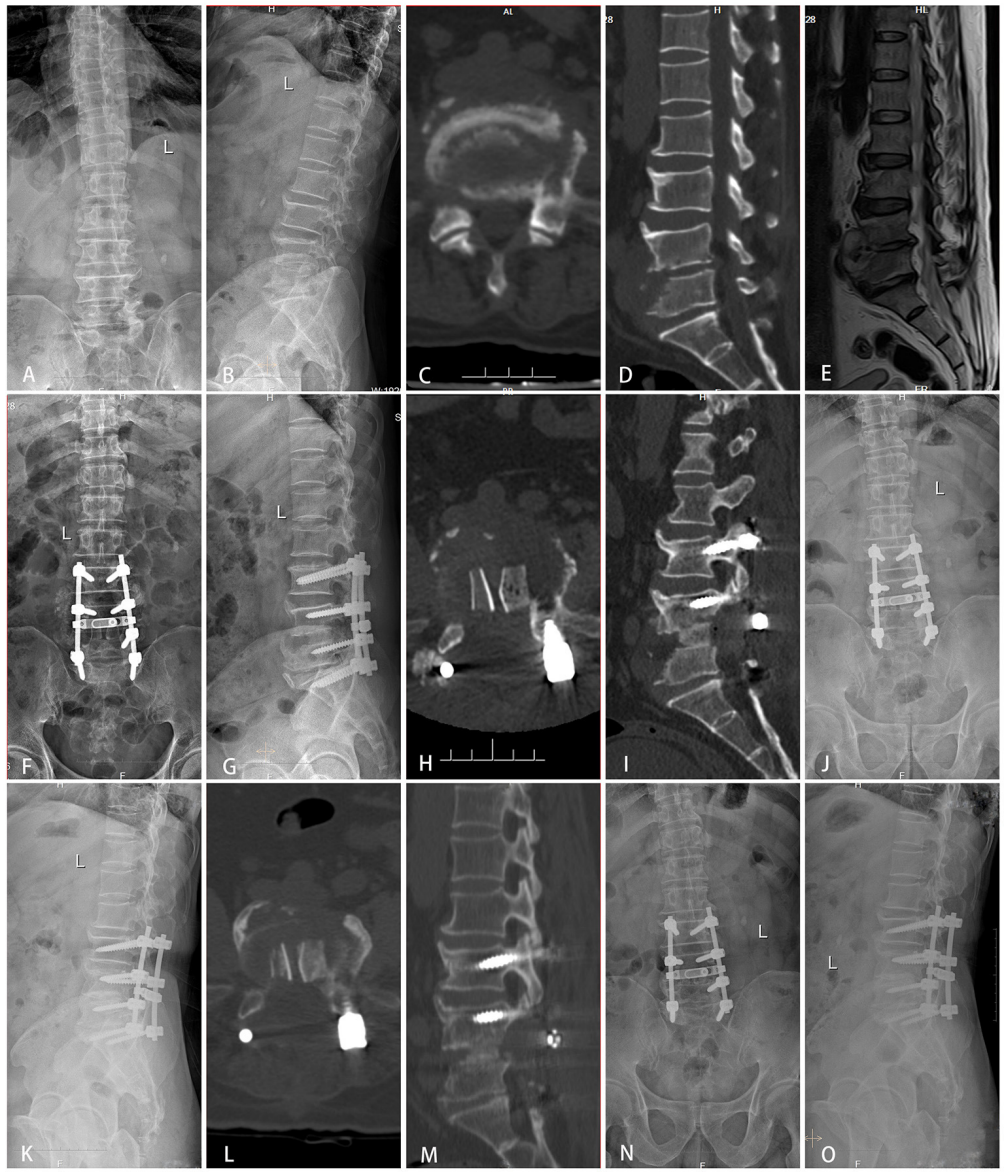
Typical cases of group C (allogeneic bone graft). A 55-year-old male was diagnosed with tuberculous spondylitis after a one-year history of severe low back pain. The infection had been resistant to chemotherapy for 3 months. **(A–E)** Preoperative X-ray, MRI and CT showed that the lesion around the vertebral body of L4/5 developed an abscess with marked bony destruction. **(F–I)** Postoperative X-ray and CT showed complete resolution of the epidural abscess and decompression of the neural component. Interbody grafts using two allogeneic bones were placed satisfactorily. **(J–M)** One-year follow-up showed good bone fusion. **(N,O)** Final follow-up (3 years) radiographs showed good bone fusion and no obvious bone absorption or fractures.

A total of 69 patients were divided into three groups: group A (TMC+SP bone graft: 12 patients, Figure 1), group B (TMC bone graft: 30 patients, Figure 2) and group C (allogeneic bone graft: 27 patients, Figure 3). The mean follow-up times were 35.29 ± 6.69 months, 34.57 ± 6.65 months and 35.1 ± 6.46 months, respectively (*p* = 0.92). No significant differences were observed in sex (*p* = 0.57), age (*p* = 0.58), ODI (*p* = 0.87), ODI-FFU (*p* = 0.80), VAS score (*p* = 0.72), VAS-FFU (*p* = 0.78), or hospital stay (*p* = 0.54) (Tables 1, 2). No significant difference in ESR or CRP was found at any time point (Supplementary Table 1). A total of 74–78% of patients were from rural areas, while 67–74% of patients were farmers and workers. Regarding income, 87–92% of patients had an annual individual income < $5,000 (Table 1).

**Table 1:** Demographics of study populations.

**Clinical features**	**Group A (***n*** = 12)**	**Group B (***n*** = 30)**	**Group C (***n*** = 27)**	***P*** **value**
Age (yr.)	48.52 ± 14.32	50.4 ± 13.20	46.67 ± 15.06	0.62	P_AB_ = 0.52 P_AC_ = 0.84 P_BC_ = 0.33
Male sex (no. [%])	6 (50%)	15 (50%)	17 (63%)	0.51	P_AB_ = 0.87 P_AC_ = 0.29 P_BC_ = 0.33
Residence (no. [%])					
Rural	9 (75%)	22 (74%)	21 (78%)		
Urban	3 (25%)	8 (26%)	6 (22%)	0.87	
Occupation					
Farmer	6 (50%)	16 (53%)	15 (56%)		
Worker	2 (17%)	6 (20%)	5 (18%)		
Student	1 (8%)	3 (10%)	3 (11%)		
Others	3 (25%)	5 (17%)	4 (15%)	1	
Annual individual income (US)				
< $2000	1(8%)	5 (17%)	5 (18%)		
$2,000–$4,999	9 (75%)	21 (70%)	18 (67%)		
≥$5,000	2 (17%)	4 (13%)	4 (15%)	0.98	
Hospital cost (US)	$14,710.42 ± 2,354.55	$16,680.23 ± 3,614.73	$19,260.34 ± 33,100.75	0.00	P_AB_ = 0.03 P_AC_ <0.01 P_BC_ <0.01
Hospital stays (day)	24.71 ± 8.85	26.20 ± 5.95	26.89 ± 5.31	0.54	P_AB_ = 0.48 P_AC_ = 0.30 P_BC_ = 0.65
Duration of follow-up (months)	35.29 ± 6.69	34.57 ± 6.65	35.15 ± 6.46	0.92	P_AB_ = 0.71 P_AC_ = 0.94 P_BC_ = 0.74

**Table 2:** Clinical data of study populations.

**Clinical features**	**Group A (***n*** = 12)**	**Group B (***n*** = 30)**	**Group C (***n*** = 27)**	***p*** **value**
Infected spinal level				
L1–2	2	3	4		
L2–3	3	7	3		
L3–4	2	5	6		
L4–5	4	9	10		
L5–S1	1	6	4		
Fixation Segment	1.67 ± 0.64	1.83 ± 0.90	2.81 ± 0.94	0.00	P_AB_ = 0.47 P_AC_ <0.01 P_BC_ <0.01
Number of pedicle screw	5.05 ± 1.29	5.53 ± 1.73	6.85 ± 1.37	0.00	P_AB_ = 0.28 P_AC_ <0.01 P_BC_ <0.01
ODI	0.75 ± 0.16	0.73 ± 0.12	0.73 ± 0.11	0.87	P_AB_ = 0.62 P_AC_ = 0.69 P_BC_ = 0.89
ODI-FFU	0.18 ± 0.05	0.19 ± 0.04	0.19 ± 0.06	0.80	P_AB_ = 0.68 P_AC_ = 0.54 P_BC_ = 0.73
VAS	7.05 ± 1.53	7.27 ± 1.46	6.96 ± 1.26	0.72	P_AB_ = 0.61 P_AC_ = 0.83 P_BC_ = 0.41
VAS-FFU	1.38 ± 0.84	1.53 ± 0.76	1.52 ± 0.79	0.78	P_AB_ = 0.51 P_AC_ = 0.57 P_BC_ = 0.94
Operation blood loss (ml)	543.81 ± 230.81	584.00 ± 229.06	803.70 ± 446.78	0.01	P_AB_ = 066. P_AC_ <0.01 P_BC_ = 0.01
Operation time (min)	166.43 ± 44.11	189.00 ± 41.64	205.93 ± 51.73	0.02	P_AB_ = 0.1 P_AC_ <0.01 P_BC_ = 0.19
Duration of follow-up (months)	35.29 ± 6.69	34.57 ± 6.65	35.15 ± 6.46	0.92	P_AB_ = 0.71 P_AC_ = 0.94 P_BC_ = 0.74

*FFU, Final follow-up*.

Regarding the number of fixation segments and pedicle screws, both groups A and B had significantly fewer fixation segments and pedicle screws than group C (*p* < 0.001), while no significant difference was found between groups A and B (*p* > 0.01, Table 2). Consequently, the hospital costs of group A and group B were lower than that of group C ($14,710.42 ± 2,354.55 vs. $16,680.23 ± 3,614.73 vs. $19,260.34 ± 3,310.75, *p* < 0.01; P_AC_ < 0.01 P_BC_ < 0.01, respectively, Table 1). There was a significant difference in hospital cost between groups A and B (P_AB_ = 0.03). In terms of operative time, a significant difference was observed among all three groups (*p* = 0.02), with group A (166.43 ± 44.11 min) having a shorter operative time than group C (205.93 ± 51.73 min, *p* < 0.01). There was no significant difference between groups A and B (*p* = 0.1) or between groups B and C (*p* = 0.19) (Table 1). There was a significant difference in blood loss among the three groups (543.81 ± 230.81 ml vs. 584.00 ± 229.06 ml vs. 803.70 ± 446.78 ml; *p* = 0.01, P_AB_ = 066. P_AC_ < 0.01 P_BC_ = 0.01) (Table 1).

No significant difference was observed in the preoperative, postoperative, or final follow-up Cobb angles among groups A, B, and C (*p* = 0.99, 0.71 and 0.99). Moreover, there was no significant difference in Cobb angle correction or loss among the three groups (*p* = 0.88 and 0.98). The pelvic parameters (PT, PI, SS) of the three groups were not significantly different at any time point (Supplementary Table 2). The LL of the three groups was not significantly different at the preoperative, postoperative, or final follow-up (*p* = 0.94, 0.78 and 0.81, respectively). In addition, the LL correction and loss among the three groups were not significantly different (*p* = 0.68 and 0.33, respectively). Similarly, the PI-LL of the three groups showed no significant difference in preoperative, postoperative, final follow-up or loss of correction parameters (*p* = 0.38, 0.19, 0.14 and 0.23, respectively). There was no significant difference in bone graft fusion time among the three groups (8.90 ± 2.11 months vs. 8.60 ± 2.39 months vs. 9.59 ± 2.04 months, *p* = 0.25) (Supplementary Table 2).

With respect to neurological status, the ASIA grade showed no difference among the three groups before surgery (*p* = 0.88) or at the last follow-up (*p* = 0.957) (Table 3). As shown in Table 4, there were no significant postoperative complications among the three groups (*p* = 0.81), and all patients were cured after active treatment.

**Table 3:** The neurological function evaluated by the ASIA impairment scale.

**ASIA scale**	**Group A**	**Group B**	**Group C**	***P*** **value**
	**(***N*** = 12)**	**(***N*** = 30)**	**(***N*** = 27)**	
**Pre**	**Pre**	**Pre**		
A	0	0	0	0.883
B	0	0	0	
C	2	5	3	
D	4	12	10	
E	6	13	14	
**FFU**	**FFU**	**FFU**	0.957	
A	0	0	0	
B	0	0	0	
C	0	2	0	
D	2	3	2	
E	10	25	25	

*Pre, Preoperation; FFU, Final follow-up*.

**Table 4:** Comparison of postoperative complications of study populations.

**Complications**	**Group A**	**Group B**	**Group C**	***P*** **value**
		**(***N*** = 12)**	**(***N*** = 30)**	**(***N*** = 27)**	
Systemic complications				
Pulmonary infection	1	2	2	
Hepatic dysfunction	1	1	2	
Renal dysfunction	1	3	2	
Urinary tract infection	1	0	2	
Deep vein thrombosis	0	2	1	
Local complications				
Cerebrospinal fluid linkage	0	1	1	
Sinus formation	1	2	2	
TMC dislocation	0	2	0	
Bone graft absorbed	0	0	1	
Total	5	13	14	0.805

## Discussion

STB often causes damage in the anterior and middle column of the spine, leading to vertebral destruction, abscess formation, angular deformation, and neurological dysfunction (2). Surgical intervention plays an important role in lesion debridement, decompression, and spinal stability reconstruction, which is beneficial to treat STB and to prevent recurrence (16). Three main surgical approaches for treating lumbar TB exist: the anterior approach, posterior approach, and the posterior combined anterior approach. The anterior approach allows the surgeon to directly focus on implanting the bone graft; however, it has a disadvantage in correcting kyphosis and preventing correction loss (17). Considering this defect, the posterior combined anterior approach has been applied to enhance kyphosis correction and prevent correction loss and graft failure. However, this combined approach requires a longer operation time, greater surgical trauma, and longer recovery times (18). The posterior-only approach seems to be a better choice, as numerous studies have reported that the posterior-only approach can safely and effectively achieve the same clinical results as the posterior combined anterior approach but with less trauma, lower cost, and fewer complications (19). However, lesions are mainly in the anterior and middle columns, which requires surgeons to perform lesion debridement and reconstruct spine stability (20).

According to the 3-column theory of Denis et al. (21), integrating the anterior column and the middle column is of key importance for the reconstruction of spinal stability. For interbody fusion in patients with tuberculosis spondylitis, autogenous iliac bone has long been considered the best method since it results in good osteogenesis, bone induction, bone conductibility, and biocompatibility (13). However, the preparation of autogenous iliac bone prolongs the operative time, increasing trauma and the risk of donor site complications. It has been reported that up to 40% of cases suffer from chronic pain and wound infection (22). There is also a risk of bone absorption (23). Allogeneic iliac bone may cause a mild chronic inflammatory reaction, which slows the formation and growth of blood vessels and interferes with osteoclast and osteoblast remodeling on the bone contact surface. The bone fusion time is relatively longer than that of autologous bone (24, 25). Previous studies reported that TMCs provide better structural support for kyphosis and intervertebral height correction than autogenous iliac bone, and they are immune to the degradative enzymes that reside in an infected environment. However, a TMC has a risk of subsiding or displacement, which is related to the contact area, bone strength, and surgery (26, 27). Recently, several authors have reported on the use of SP bone for the treatment of spinal infection (12, 13, 28). Zhong et al. (12) reviewed 35 cases treated with SP bone in one-level thoracic or lumbar tuberculosis and found that the mean bone fusion time was 12.90 ± 3.91 months. Tang (13) compared SP, transverse process (TP) and iliac bone grafts in single-segment thoracic tuberculosis, and the mean bone fusion times were 12.90 ± 3.91 months, 6.75 ± 1.55 months, and 5.52 ± 1.64 months, respectively. According to their reports, the use of the SP could be suitable for strutting the bone defect space, representing an additional choice for surgeons in segmental stability construction. However, because using a single SP as a bone graft conveys a risk of delayed bony fusion or even nonunion, the author suggested prolonged brace treatment.

In our study, we chose SP combined with a TMC for reconstruction of the anterior and middle columns of the spine. Usually, we first implant the shaped SP followed by the TMC (filled with autogenous cancellous bone granules). Finally, we tightened the titanium rod with proper pressure and confirmed that the TMC and SP were in good positions. Since this is the first report on one-level lumbar and lumbosacral STB treated with SP+TMC methods, we compared it to TMCs (group B) and allografts (group C) regarding three aspects: safety, efficacy, and cost-effectiveness.

### Safety

There were 12 patients (group A, Figure 1) who underwent SP+TMC bone grafts with a significant improvement in the VAS score and ODI at the FFU, at which time CRP and ESR had returned to normal. All patients achieved bone fusion at a mean time of 8.90 ± 2.11 months, and all patients with neurological defects were improved at the FFU, indicating that the STB was cured. Moreover, there was no significant difference in postoperative complications compared to other groups. The above data indicate the safety of SP+TMC graft methods in lumbar and lumbosacral STB surgery.

### Efficacy

Our study found that SP+TMC (group A) exhibited fewer fixation segments, fewer pedicle screw implants (5.05 ± 1.29 vs. 6.85 ± 1.37 P_AC_ < 0.01), shorter operation times (166.43 ± 44.11 min vs. 205.93 ± 51.73 min P_AC_ < 0.01), and reduced intraoperative blood loss (543.81 ± 230.81 ml vs. 803.70 ± 446.78 ml P_AC_ < 0.01) compared to the allograft (group C, Figure 3). The underlying reason for this phenomenon could be that because allogeneic iliac bone has a weaker osteoinduction ability, surgeons tend to choose a more stable fixation scheme, i.e., lengthening the fixed segment when using allogeneic iliac bone in bone fusion. The postoperative and FFU radiological assessments between the two groups showed no significant difference in Cobb angle or LL correction and maintenance, while the pelvic parameters and PI-LL showed no obvious sagittal imbalance in any group. Although there was no significant difference in bone graft fusion time among the three groups (8.90 ± 2.11 months vs. 8.60 ± 2.39 months vs. 9.59 ± 2.04 months, *p* = 0.25) (Supplementary Table 2), compared to previous reports, the SP+TMC group had a significantly shorter time of bone fusion than the SP-only group (12, 13). Moreover, the postoperative and follow-up data showed that the SP+TMC group achieved the same satisfying clinical results in relatively short segment fixation compared to the allograft group with long segment fusion.

### Cost-Effectiveness

In the past 5 years, the global total budget for TB has continually increased, reaching $994 million USD in 2020, and the rapid increase in the TB budget has caused a heavy economic burden to society (1). The average annual disposable income per person is approximately $2,000 in rural and urban areas and $5,000 in our areas. In our study, 74%-78% of patients came from rural areas. Regarding careers, 67%-74% of patients were farmers and workers with insufficient health insurance. A total of 87%-92% of patients had an annual individual income of < $5,000. For these people, it is of great significance to reduce the cost of treatment on the premise of ensuring the safety and efficacy of the operation. Since the mean hospital cost was $14,710.42 ± 2,354.55 in group A, $16,680.23 ± 3,614.73 in group B (Figure 2), and $19,260.34 ± 33,100.75 in group C, there was a significant decrease in hospital cost in group A compared to groups B (*p* = 0.03) and C (*p* < 0.01). SP+TMC provides a method with high cost-effectiveness for patients in developing countries and areas. The reduction in cost is primarily due to the decrease in the fixation segment, the reduced number of pedicle screws and the use of allogeneic bone and titanium mesh cages.

From the above comparisons, we found that for single-segment lumbar and lumbosacral STBs, TMCs reduce the fixed segments and achieve the same effect as long segment fixation combined with allogeneic bone grafts. Additionally, the combination of SP bone reduces the cost of hospitalization. The reasons for these observations could be as follows: (1) A TMC provides immediate stability, and its rigid characteristics can tolerate compression forces well. (2) A TMC can be tailored to fit the bone graft area, increasing the contact area and weight-bearing surfaces. (3) SP, as an autogenous bone graft, has advantages with respect to osteogenesis, bone healing, bone conduction, and osteoinduction. (4) SP is present in the surgical exposure area in the posterior approach, which can reduce time, bleeding, and trauma for allogeneic iliac bone. (5) The SP, a cortical bone, has improved structural integrity and can effectively fill the defect space.

The indications for SP combined with TMCs are as follows: (1) One segment needed surgical intervention, or multiple segments were involved, but only one level needed surgical intervention. (2) Spinous process bone was not contaminated by tuberculous abscesses. (3) There is no severe osteoporosis because it may lead to the deterioration of the bone strength of the spinous process and osteogenic ability.

### Limitations of the Study

First, this study did not consider intra- or interobserver differences associated with bias. Second, the retrospective nature of the study and small sample size may have introduced bias.

## Conclusion

Our study revealed that compared to TMC and allograft treatment, SP combined with a TMC as a bone graft may represent an effective and cost-effective approach for the surgical management of one-level lumbar or lumbosacral spinal TB, leading to the effective restoration of spinal stability. This approach is a reliable structural bone grafting method, especially for people living in developing countries or rural areas.

## Data Availability Statement

The raw data supporting the conclusions of this article will be made available by the authors, without undue reservation.

## Ethics Statement

The studies involving human participants were reviewed and approved by the Medical Ethics Committee of Xiangya Hospital, Central South University (Ethical Code: 201703358). The patients/participants provided their written informed consent to participate in this study.

## Author Contributions

HZ designed the study. LX and MT performed the data collection, statistical analysis, and data interpretation. GY contributed to manuscript writing. GY and LX contributed to patient enrolment and follow-up. All authors read and approved the final manuscript.

## Funding

This study was supported by the National Natural Science Foundation of Hunan (2019JJ80014). No benefit in any form has been or will be received from a commercial party related directly or indirectly to the subject of this manuscript.

## Conflict of Interest

The authors declare that the research was conducted in the absence of any commercial or financial relationships that could be construed as a potential conflict of interest.

## Publisher's Note

All claims expressed in this article are solely those of the authors and do not necessarily represent those of their affiliated organizations, or those of the publisher, the editors and the reviewers. Any product that may be evaluated in this article, or claim that may be made by its manufacturer, is not guaranteed or endorsed by the publisher.
