# Supplementary material for: Spinous Process Combined With a Titanium Mesh Cage as a Bone Graft in the Stability Reconstruction of Lumbar or Lumbosacral Spinal Tuberculosis
Source: Front Surg. 2022 Apr 4;9:818926. doi: 10.3389/fsurg.2022.818926 (PMC9013749; doi:10.3389/fsurg.2022.818926)
Supplement: Supplementary file 1 [file Table_1.DOCX]

| **Supplemental Table I** laboratory examination of study populations | | | | | | | | |
| --- | --- | --- | --- | --- | --- | --- | --- | --- |
| Clinical features | | Group A (n=12) | Group B (n=30) | Group C (n=27) | p value | | | |
| Mean value of pre-operation | | | |  |  |  |  |  |
|  | ESR (mm/h) | 67.57 ±28.87 | 71.33 ±28.60 | 60.33 ±27.00 | 0.35 | P_AB_=0.65 P_AC_=0.38 P_BC_=0.14 | | |
|  | CRP (mg/l) | 45.67 ±33.02 | 50.20 ±26.35 | 45.71 ±21.79 | 0.78 | P_AB_=0.59 P_AC_=0.99 P_BC_=0.49 | | |
| Mean value of post-operation | | | |  |  |  |  |  |
|  | ESR (mm/h) | 60.62 ±28.39 | 65.90 ±29.11 | 59.56 ±33.73 | 0.72 | P_AB_=0.53 P_AC_=0.91 P_BC_=0.45 | | |
|  | CRP (mg/l) | 21.28 ±29.79 | 26.87 ±28.38 | 19.82 ±18.42 | 0.57 | P_AB_=0.50 P_AC_=0.83 P_BC_=0.28 | | |
| Mean value of Final follow-up | | | |  |  |  |  |  |
|  | ESR (mm/h) | 12.62 ±3.97 | 12.50 ±4.19 | 11.48 ±4.15 | 0.57 | P_AB_=0.92 P_AC_=0.35 P_BC_=0.36 | | |
|  | CRP (mg/l) | 4.43 ±2.04 | 4.90 ±1.90 | 4.96 ±2.00 | 0.62 | P_AB_=0.41 P_AC_=0.38 P_BC_=0.91 | | |

Abbreviation: FFU Final follow-up
