# Supplementary material for: Spinous Process Combined With a Titanium Mesh Cage as a Bone Graft in the Stability Reconstruction of Lumbar or Lumbosacral Spinal Tuberculosis
Source: Front Surg. 2022 Apr 4;9:818926. doi: 10.3389/fsurg.2022.818926 (PMC9013749; doi:10.3389/fsurg.2022.818926)
Supplement: Supplementary file 2 [file Table_2.DOCX]

| **Supplemental Table 2** Radiographic assessments of study populations | | | | | |
| --- | --- | --- | --- | --- | --- |
| Radiographic data | | Group A (n=12) | Group B (n=30) | Group C (n=27) | P value |
| Cobb angle (°) | | | | | |
|  | Pre-operative | 13.43±7.62 | 13.06 ±9.07 | 13.07 ±7.58 | 0.99 |
|  | Postoperative | 11.33 ±8.85 | 11.84 ±10.46 | 13.89 ±9.86 | 0.71 |
|  | Last follow-up | 11.42 ±8.38 | 11.90 ±10.24 | 11.39 ±8.59 | 0.99 |
|  | Mean Cobb angle correction | 6.46 ±4.45 | 6.70±5.06 | 7.27±5.17 | 0.88 |
|  | Mean Correction loss | 3.38±3.90 | 3.14±3.83 | 3.33±3.26 | 0.98 |
| Lumbar lordosis (°) | | | | | |
|  | Pre-operative | 37.71±13.12 | 39.61±12.63 | 38.20±15.97 | 0.94 |
|  | Postoperative | 39.11±9.94 | 41.29±10.18 | 38.57±12.36 | 0.78 |
|  | Last follow-up | 39.12±11.35 | 42.02±9.50 | 39.82±14.04 | 0.81 |
|  | Mean Lumbar lordosis correction | 9.98±5.33 | 7.74±6.12 | 9.22±7.89 | 0.68 |
|  | Mean Correction loss | 5.63±4.15 | 3.41±3.21 | 4.54±3.97 | 0.33 |
| PT (°) | | | | | |
|  | Pre-operative | 13.32±7.11 | 16.69±8.26 | 14.41±7.90 | 0.74 |
|  | Postoperative | 18.25±11.00 | 10.20±5.94 | 11.84±6.94 | 0.49 |
|  | Last follow-up | 14.35±8.80 | 11.26±6.27 | 13.51±7.55 | 0.55 |
|  | Mean Correction loss | 3.24±3.97 | 2.71±2.98 | 2.63±2.79 | 0.85 |
| PI (°) | | | | | |
|  | Pre-operative | 42.59±9.26 | 46.49±8.97 | 45.08±8.93 | 0.88 |
|  | Postoperative | 49.48±13.50 | 43.79±9.17 | 42.92±9.89 | 0.94 |
|  | Last follow-up | 43.18±9.87 | 43.38±8.19 | 43.50±10.43 | 1.00 |
|  | Mean Correction loss | 2.78±2.36 | 3.46±3.39 | 2.91±2.65 | 0.79 |
| SS (°) | | | | | |
|  | Pre-operative | 29.84±4.78 | 29.80±8.06 | 31.47±8.21 | 0.84 |
|  | Postoperative | 31.23±3.51 | 33.59±8.45 | 31.45±7.65 | 0.40 |
|  | Last follow-up | 28.97±6.47 | 32.12±7.12 | 30.07±8.27 | 0.54 |
|  | Mean Correction loss | 3.45±2.84 | 2.50±2.86 | 2.77±3.01 | 0.68 |
| PI-LL (°) | | | | | |
|  | Pre-operative | 10.81±7.84 | 8.86±8.30 | 13.34±9.92 | 0.38 |
|  | Postoperative | 5.97±3.94 | 6.36±4.89 | 8.62±4.97 | 0.19 |
|  | Last follow-up | 7.37±4.60 | 5.29±4.76 | 8.90±5.61 | 0.14 |
|  | Mean Correction loss | 3.53±2.98 | 1.82±2.06 | 2.73±2.53 | 0.23 |
| Bone graft fusion time (months) | | 8.90 ±2.11 | 8.60±2.39 | 9.59 ±2.04 | 0.25 |
